# Extensive electronic investigation of BMBH structure and adsorption locator on graphene with molecular dynamics of human serum albumin interaction

**DOI:** 10.1038/s41598-025-04180-4

**Published:** 2025-06-20

**Authors:** Walaa S. S. Alblozy, Doaa S. El Sayed, Refaat M. Mahfouz

**Affiliations:** 1 Chemistry Department, Faculty of Science, Assiut University, Assiut, Egypt; 2 Chemistry Department, Faculty of Science, Alexandria University, Baghdad Street, Moharam Bey, P.O. Box 21511, Alexandria, Egypt

**Keywords:** BMBH drug, Computational study, DFT calculations, Graphene adsorption, Molecular docking, Dynamic simulation, Theoretical chemistry, Computational chemistry, Structural materials

## Abstract

A comprehensive electronic investigation of Bambuterol Hydrochloride (BMBH) was conducted to explore its structural properties, adsorption behavior on graphene, molecular docking interactions, and molecular dynamics perturbations. FT-IR and XRD characteristics were performed to support the structural identity. Geometry optimization and theoretical calculations were carried out to study the structural and electronic properties of BMBH. The nature of hydrogen and halogen bonding interactions was analyzed using natural bond orbital (NBO) analysis, atoms in molecules (AIM) theory, and Reduced Density Gradient (RDG) analysis. Additionally, electron localization function (ELF) analysis provided deeper insights into the chemical bonding characteristics of BMB. Adsorption locator modelling was involved to allow activated carbon-carriers for sustained and controlled drug release, which helps maintain therapeutic drug levels in the body over time, reducing the frequency of administration. Molecular docking analysis was performed to assess the interaction of BMBH with key biological targets, revealing its potential pharmacological relevance. The inhibitory interaction of BMB with the butyrylcholinesterase enzyme, which is a major cause of dementia and Alzheimer’s disease, has been investigated based on molecular modelling. In addition to that the interaction between BMB and Human Serum Albumin (HSA) was assessed using molecular Docking and Molecular dynamics studies to investigate its transportation and bioavailability. Additionally, molecular dynamics simulations were employed to evaluate the structural perturbations and dynamic behaviour of the BMBH/graphene and BMB/target complexes over time. The study offers a detailed understanding of the electronic and interactional properties of BMB, contributing to its potential applications in nanomaterial-based drug delivery and therapeutic interventions.

## Introduction

**Fig. 1:**
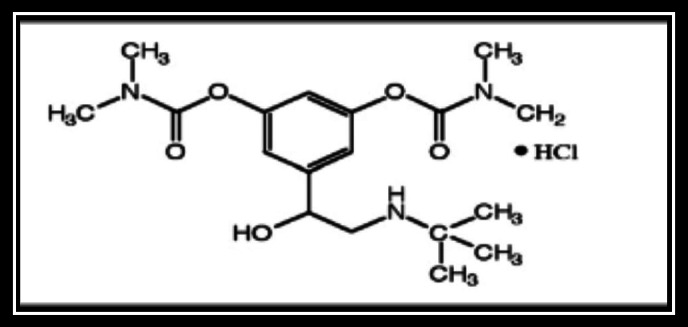
The molecular structure of 5-(2-(tert-butylamino)-1-hydroxyethyl)-1,3-phenylene bis(dimethylcarbamate) hydrochloride (BMBH).

The primary goal of scientists and dosage form designers is to improve the safety of a drug molecule while preserving its therapeutic efficacy^1–3^. Recent advancements in Novel Drug Delivery Systems (NDDS) aim to achieve this by developing dosage forms that are easy to administer, ultimately improving patient compliance. Pharmaceutical technologists have focused their efforts on creating Fast Dissolving Drug Delivery Systems^4–6^. These tablets, which rapidly disintegrate or dissolve in the mouth, are especially beneficial for children, elderly patients, those with swallowing difficulties, or when drinking liquids may not be feasible. Bambuterol hydrochloride is a long-acting β₂-adrenergic receptor agonist (LABA) used primarily in managing asthma and chronic obstructive pulmonary disease (COPD)^7,8^. As a prodrug of terbutaline, it undergoes hepatic metabolism to release the active compound, providing a sustained bronchodilatory effect. Due to its extended half-life, bambuterol offers advantages over short-acting β₂-agonists by reducing dosing frequency and improving patient compliance^9^. This research examines bambuterol hydrochloride in pulmonary medicine. It highlights its potential to improve disease control, patient adherence, and treatment outcomes for asthma and COPD. Bambuterol hydrochloride (BMBH), chemically known as (RS)-5-(2-tert-butylamino-1-hydroxyethyl)-m-phenylene bis(dimethyl carbamate) hydrochloride (Fig. 1), is a long-acting, orally administered sympathomimetic drug with predominant adrenergic activity (β1-agonist). It is widely prescribed for asthma and COPD management^10–12^. As an ester prodrug of the β2 adrenergic agonist terbutaline^13,14^, bambuterol hydrochloride has been analyzed using solid-state NMR spectroscopy, GC–MS, and potentiometric titration. Research suggests that its active enantiomer is more effective in treating asthma than the (S)-bambuterol hydrochloride enantiomer^15^. It acts as a directly acting sympathomimetic agent and primarily exhibits adrenergic activity^16^. As a dicarbamate ester, it remains stable pre-systemically until it reaches lung tissues, where it undergoes hydrolysis by butyrylcholinesterase to release terbutaline, the active β2 adrenergic agonist^17^. Applying NDDS such as SEDDS, SMEDDS, and FDTs for bambuterol hydrochloride has shown promise in addressing its bioavailability challenges, potentially leading to more effective asthma management strategies. SEDDS enhance drug solubility and bioavailability by self emulsifying in gastrointestinal fluids. A study formulated a solid SEDDS for BMBH, showing improved permeability and potential bioavailability. SMEDDS forms microemulsions for better drug absorption. BMBH/ SMEDDS showed stability, rapid emulsification, and enhanced permeability, suggesting improved bioavailability. FDTs dissolve quickly for faster action and better compliance. BMBH/FDT with crospovidone showed the best disintegration and dissolution, suggesting improved bioavailability. NDDS like SEDDS, SMEDDS, and FDTs offer promise for better asthma treatment^18,19^.

In this study, density functional theory (DFT) calculations have been employed as a robust theoretical approach to complement the experimental characterization of bambuterol hydrochloride (BMBH) and to enhance understanding of its in-vivo pharmacological interactions. Adsorption on Graphene system can give an insight into the stability of BMBH with enhancement hydrogen attraction and storage on the electron rich surface. Investigations include molecular geometry analysis, natural bond orbital (NBO) analysis, hydrogen bonding interactions, as well as AIM, ELF, RDG, and FT-IR spectroscopy studies (Fig. 1).

## Materials and methods

All the chemicals used were of analytical grade. Spectroscopic-grade solvent ethanol (EtOH), and 5-(2-(tert-butylamino)-1-hydroxyethyl)-1,3-phenylene bis(dimethylcarbamate) hydrochloride (bambuterol hydrochloride) (purity ≥ 98%), were sourced from Sigma-Aldrich. Fourier transform infrared spectroscopy (FT-IR) was used to detect the functional groups of samples from 400–4000 cm^−1^ using a Perkin Elmer spectrometer via the KBr pellet method (Spectrum One, USA). A T80 + UV/Vis double-beam spectrophotometer from PG Instruments Ltd, UK, recorded electronic absorption spectra within the 200–600 nm wavelength range. A high-resolution scanning electron microscope (HRSEM) was formfitting with EDX (JEOL, JSM [model no: 6360]). X-ray diffraction (XRD) patterns were determined via Cu Kα X-ray radiation (Germany, λ = 1.540 Å, PAN analytical X’PERT PRO). The diffraction angle (θ), diffraction intensity (I), peak area (A), and full width at half maximum (FWHM) can be derived from the XRD spectrum^20^ as shown in Table 1.

**Table 1:** X-ray diffraction (XRD) parameters of BMBH, including peak position (2θ), d-spacing, intensity (cts), full width at half maximum (FWHM), and relative intensity (%).

Pos. (2θ°)	d-spacing (Å)	Height (cts)	FWHM Left (2θ°)	Rel. Int. (%)
10.9526	8.07150	1418.61	0.0568	100.00
11.8952	7.43394	110.09	0.0709	7.76
12.4730	7.09089	74.75	0.0896	5.27
12.8175	6.90103	54.59	0.0836	3.85
16.2360	5.45491	47.31	0.0999	3.33
17.5803	5.04069	132.06	0.0994	9.31
18.0453	4.91185	25.93	0.1167	1.83
20.7065	4.28619	113.99	0.1795	8.04
20.8605	4.25489	150.54	0.1041	10.61
21.5291	4.12424	47.66	0.1404	3.36
22.1105	4.01708	48.31	0.1010	3.41
22.8845	3.88294	41.24	0.1439	2.91
23.9619	3.71073	23.64	0.2168	1.67
24.8661	3.57782	115.80	0.1710	8.16
25.1781	3.53419	222.41	0.1256	15.68
26.0731	3.41486	15.88	0.2536	1.12
26.5183	3.35853	26.42	0.1565	1.86
27.4896	3.24203	24.37	0.2696	1.72
28.6422	3.11413	49.56	0.1788	3.49
30.1908	2.95783	6.71	0.1446	0.47
32.0110	2.79368	15.77	0.4180	1.11
32.6034	2.74426	22.98	0.2521	1.62
33.4553	2.67629	84.04	0.1102	5.92
33.9935	2.63514	93.18	0.1223	6.57
34.4375	2.60218	17.01	0.2186	1.20
35.6467	2.51663	46.38	0.1381	3.27
38.2024	2.35394	13.93	0.2639	0.98
43.8222	2.06422	18.19	0.2754	1.28
45.1119	2.00816	28.59	0.3087	2.02
45.5964	1.98794	26.10	0.2033	1.84
48.1462	1.88844	10.08	0.2243	0.71
49.2849	1.84744	7.81	0.3597	0.55
55.4801	1.65492	5.76	0.5923	0.41

### Computational details

DFT computations have been performed using the Gaussian 09 program package^21–23^. Geometry optimization and conformational analysis of the single molecule were performed using RB3LYP level (a hybrid approach that integrates Becke’s three-parameter exchange functional with the Lee–Yang–Parr correlation functional) with a 6–31 + G (d) basis set to achieve the optimal conditions for structural parameter calculations and ground state optimization in the gas phase. The selected DFT calculation level was achieved because it provides a good balance between accuracy and efficiency. It performs well for organic molecules, transition states, and general thermochemical properties. It accounts for electron correlation effects better than pure DFT functionals.

The dispersion-corrected DFT (DFT-D3) approach^24^ was employed in this study to give better results for non-covalent interactions^25^. The frequency calculations for the optimized structure of BMBH have shown no imaginary frequencies; hence, it confirms that they are true minima. The chemical reactivity has been predicted given the HOMO–LUMO gap and reactivity descriptors. The stability of the title compound was analyzed using natural bond order (NBO) analysis. The chemical activity was measured using molecular electrostatic potential (MEP) analysis. The activity and stability of the compound have been further investigated by the non-covalent interactions by the Multiwfn program^26^. This has been achieved through studying the atoms in molecules (AIM) approach, the electron localization function, the electrostatic potential map, and the reduced-density gradient (RDG). The Gauss View 6 program^27^ was used to enhance the visual animation to verify the normal modes.

### Molecular docking protocol

Our study examines how BMBH binds to and inhibits BChE, potentially preventing acetylcholine degradation and improving cognitive function. Molecular modelling and visualization via molecular docking were performed where the structure of Human butyrylcholinesterase in complex with thioflavine T were obtained from the RCSB Protein Data Bank (PDB ID: 6ESY) https://www.rcsb.org/structure/6esy. Docking protocol was validated by re-docking of the co-crystalized thioflavine T at the 297 active site of butyrylcholinesterase.

Another molecular docking analysis was conducted to elucidate the binding interactions between Human Serum Albumin (HSA) and BMBH. Docking against HSA helps assess drug binding, bioavailability, and potential interactions with natural compounds, influencing pharmacokinetics and efficacy. The three-dimensional structure of HSA, complexed with oxyphenbutazone, was retrieved from the Protein Data Bank (PDB ID: 2BXB)^28^ at https://www.rcsb.org/structure/2bxb. Salbutamol (Albuterol), a commonly used β₂ agonist was also docked against HAS for the sake of comparing their bioavailability including their distribution, and duration of action.

For receptors preparation, all crystallographic water molecules and co-crystallized ligands were removed using AutoDock Vina. Subsequently, polar hydrogen atoms were incorporated, and the receptor structures were subjected to energy minimization using the prepare_receptor4.py script from AutoDockTools (ADT, v1.5.6)^29,30^. Kollman-united atom charges were assigned, and the processed receptor structure was stored in PDBQT format^31^.

Ligand preparation involved the conversion of BMBH into a docking-compatible format using the prepare_ligand4.py script from ADT, followed by storage in PDBQT format. The docking grid was centred at the co-crystallized ligand-binding pocket of HSA, with dimensions set to 40 Å along each Cartesian axis (x, y, z). Docking simulations were conducted using the Lamarckian Genetic Algorithm with parameters set to 2,500,000 energy evaluations, 100 independent runs, and a population size of 150^32^. Docking conformations within 3 kcal/mol of the lowest energy pose were selected. Molecular interactions were visualized in BIOVIA Discovery Studio Visualizer 2021, generating 2D maps^33^.

### Molecular dynamics simulation

The interaction stability of BMBH within the HSA active site was further investigated through molecular dynamics (MD) simulations utilizing GROMACS-2023.1^34^. The CHARMM36 force field was implemented to parameterize the protein topology, while ligand topology parameters were derived via the CGenFF server^35,36^. To uphold periodic boundary conditions and mitigate edge effects, the system was solvated in a dodecahedral unit cell with a 10 Å buffer. Sodium and chloride ions were introduced to ensure electrostatic neutrality. Energy minimization was executed using the steepest descent algorithm with a force convergence criterion of 10.0 kJ/mol, iterating up to 50,000 steps to eliminate steric hindrances. This process was followed by two equilibration phases: the NVT (isothermal-isochoric) and NPT (isothermal-isobaric) ensembles, each conducted for 50,000 steps (10 picoseconds), employing a modified Berendsen thermostat and a leap-frog integration scheme. Subsequently, the production MD simulation was performed for 200 ns with a 2-femtosecond integration time step.

## Results and discussion

### FT-IR spectra

**Fig. 2:**
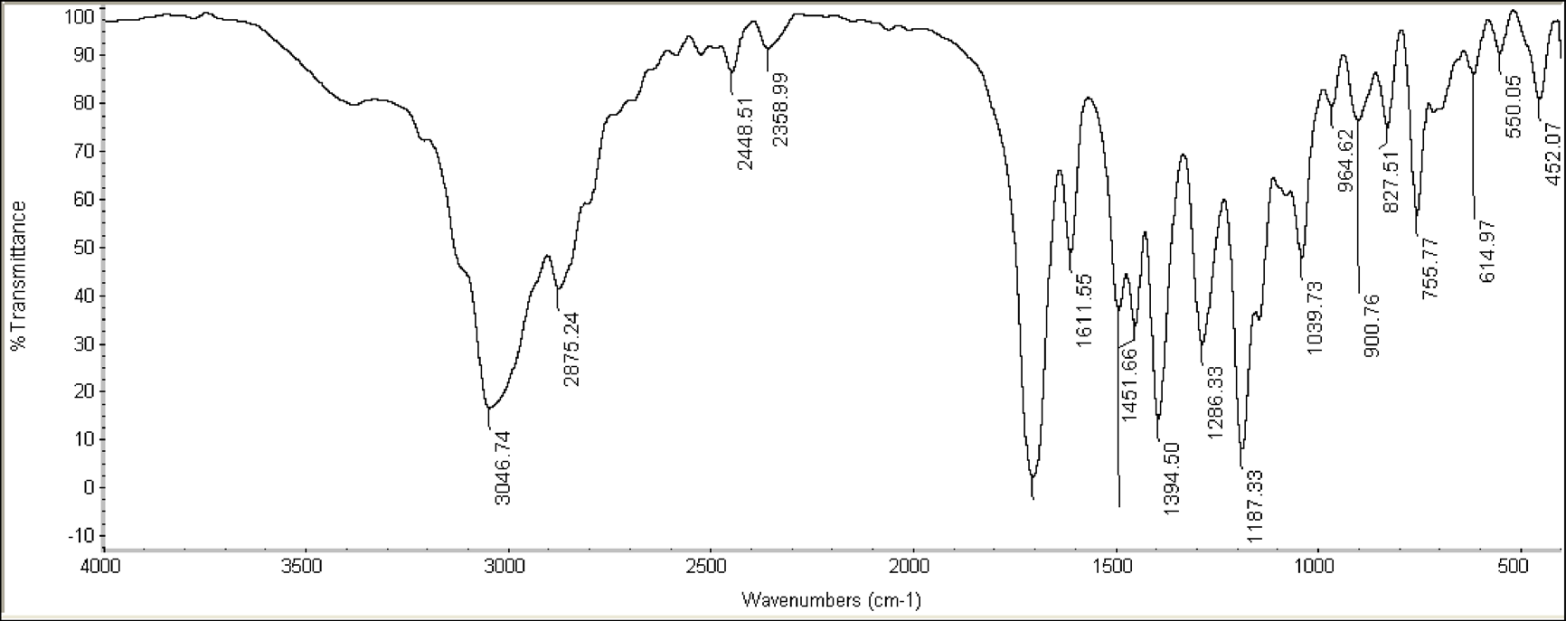
Fourier transform infrared (FT-IR) spectrum of the BMBH structure, showing characteristic absorption bands corresponding to functional groups present in the material.

The assignments were completed over the estimated range of wavenumber as shown in Fig. 2. A broadband was observed at 3046 cm^−1^, this can be attributed to the OH group. Meanwhile, the band at 2875 cm^−1^ was attributed to the N–H group of secondary amines. The later band shift may be due to the intramolecular H-bond inclusion. Other observed bands at 2448 cm^−1^ and 2358 cm^−1^ appeared for C–H aromatic and C–H aliphatic groups. A sharp band appeared at 1700 cm^−1^, was attributed to the C= O group.

The molecule consists of 57 atoms, resulting in a total of 165 vibrational modes. These modes are distributed among different symmetry species based on the C₂ point symmetry of the molecule, which has a non-planar structure. The vibrations are categorized as either in-plane or out-of-plane. The calculated wavenumbers are generally higher than the experimental values for most normal modes. This discrepancy is due to vibrational differences. Theoretical calculations use the gas phase, while experiments use the solid state. Additionally, experimental values correspond to anharmonic wavenumbers, while the computed values are harmonic. To account for this anharmonicity, a scaling factor of 0.9608 is applied to the computed wavenumbers. The methyl groups play a crucial role in the vibrational spectrum of BMBH, as the molecule contains seven methyl groups. Therefore, the assignment of methyl group vibrations is examined in detail. Each methyl group has nine vibrational modes: two asymmetric stretches, one symmetric stretch, two asymmetric deformations, one symmetric deformation, two rockings, and one torsion. The computed (scaled) vibrations of the methyl groups, along with the experimentally observed frequencies and their corresponding assignments, are summarized in Table 2.

**Table 2:** The observed FT-IR and computed frequencies atRB3LYP /6–31 + G(d) level of DFT BMBH (methyl groups vibrations).

Assignment	Exp	intensity	Scaled	computed	NO
υ_asy_ (CH3)	3002	37.36	3008	3134	1
υ_sy_ (CH3)	2898	119.99	2909	3031	2
S_asy_ (CH3)	1446	88.65	1424	1484	3
S _sy_ (CH3)	1359	105.86	1379	1437	4
_*ρ*asy_(CH3)	1140	601.4	1136	1189	5
_*ρ*sy_(CH3)	1002	62.61	1017	1064	6

ע_asy_ → asymmetric stretching.

ע_sy_ → symmetric stretching.

S_asy_ → asymmetric deformation.

S_sy_ → symmetric deformation.

_*ρ*asy_→ asymmetric rocking.

_*ρ*sy_→ symmetric rocking.

### UV–vis analysis

**Fig. 3:**
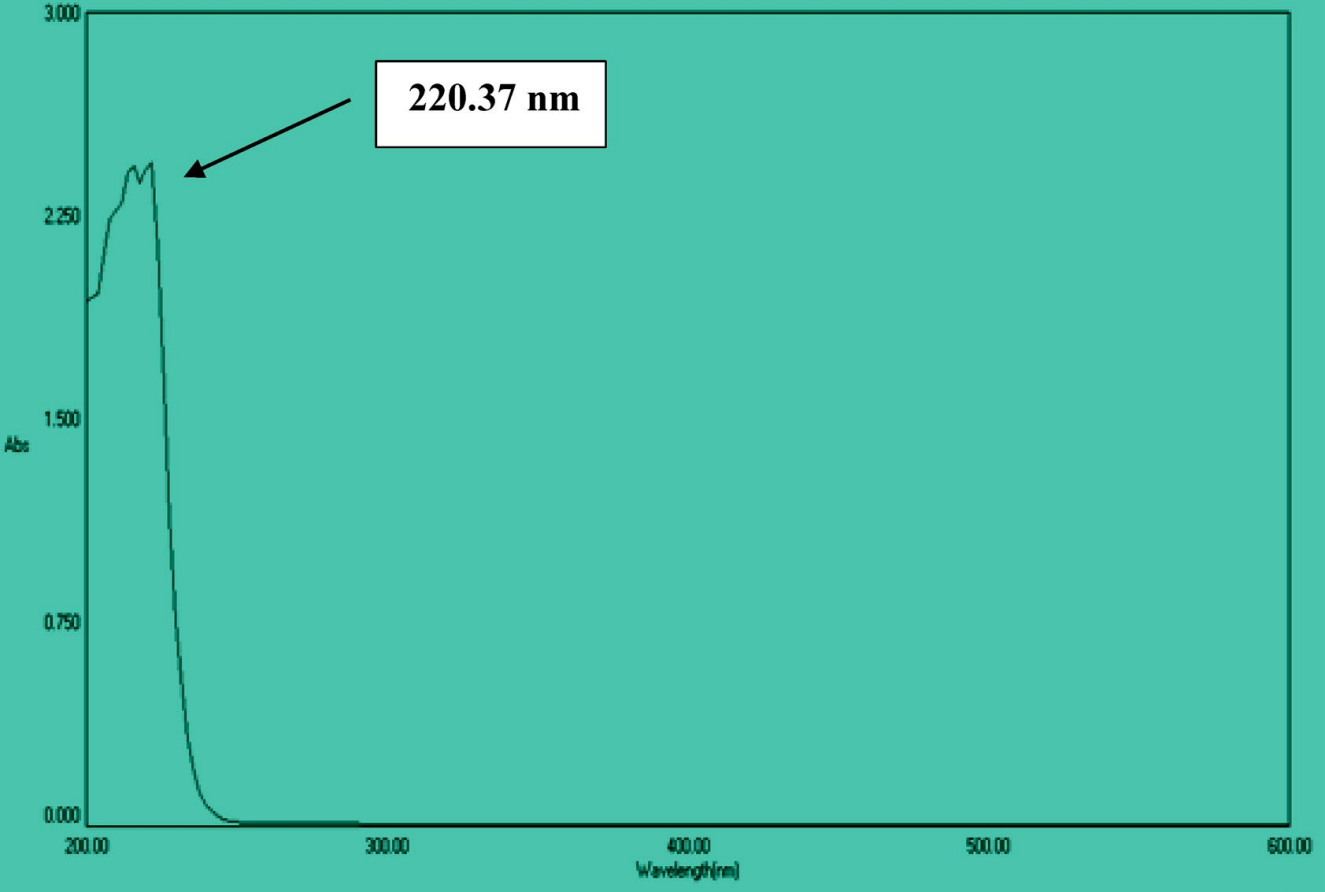
UV–vis spectrum of BMBH, displaying the absorbance characteristics of the material.

UV–Vis spectrophotometry is essential for pharmaceutical analysis, as most drugs absorb light between 200 and 800 nm. The Beer-Lambert law was applied to quantify BMBH by measuring its absorbance. Figure 3 presents the absorption spectra of BMBH, where its light absorption and transmission characteristics were initially determined experimentally using ethanol as a solvent. The experimentally observed spectrum exhibited a maximum absorption wavelength (λ_max_) of 220.37 nm in ethanol with an absorbance value equal to 2.474.

### XRD analysis

**Fig. 4:**
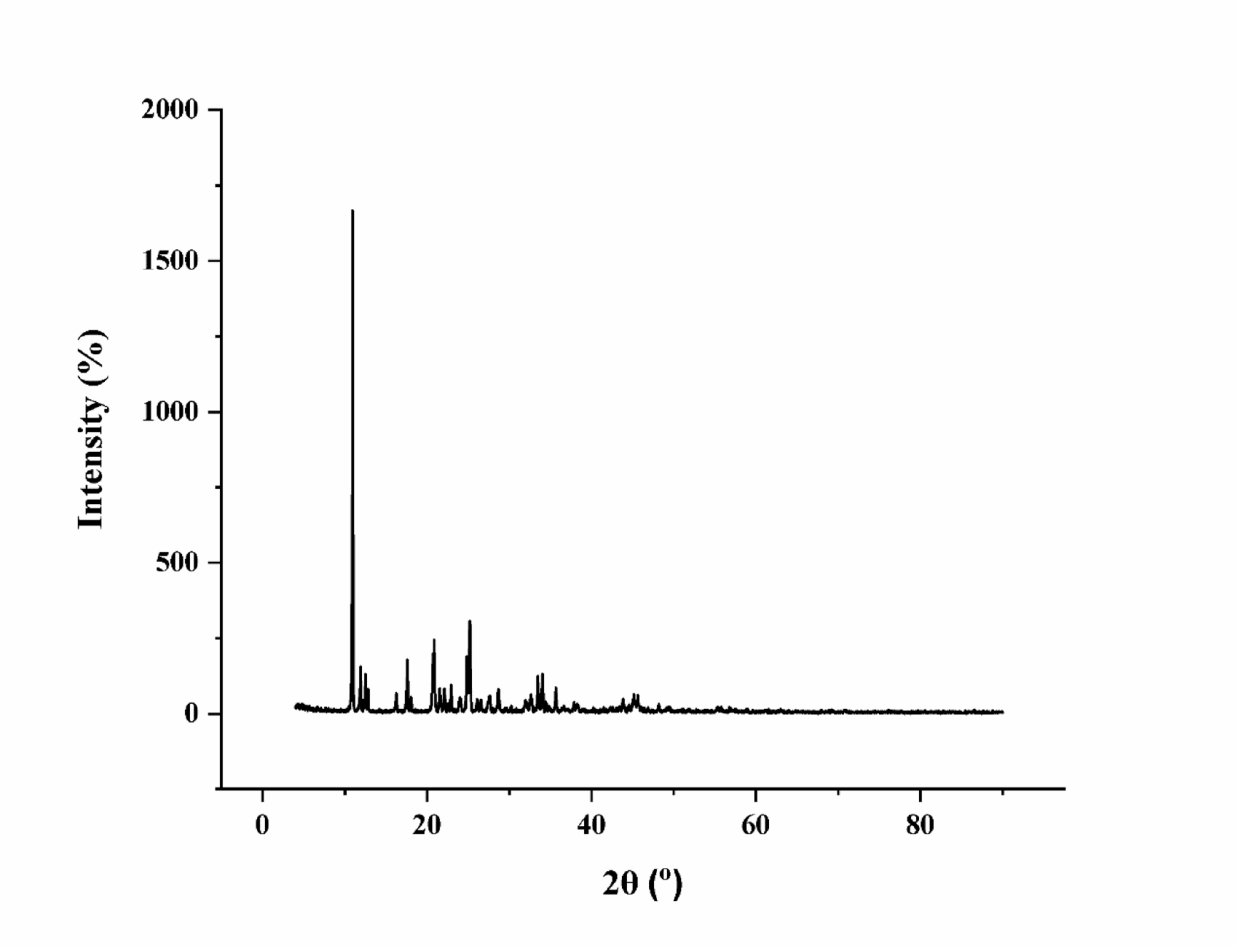
The X-ray diffraction (XRD) spectrum of BMBH illustrates the crystalline structure through diffraction peak positions and intensities.

X-ray Diffraction (XRD) is used to analyze the crystalline structure of BMBH and detect any perturbations caused by physical, chemical, or environmental factors. Perturbations in XRD patterns indicate changes in crystallinity, phase transitions, polymorphism, or interactions with excipients in pharmaceutical formulations. BMBH, in its crystalline form, exhibits sharp and well-defined diffraction peaks at characteristic 2θ angles, which correspond to its crystal lattice parameters. XRD analysis was used to show the synthesized complex’s surface structural features and characteristics^37^. Figure 4 shows the results of observed peaks, where a sharp with high-intensity peak appears at 2θ of 10°. This high diffraction spectrum intensity refers to the studied compound’s significant crystallinity.

### Computational studies

#### Geometry optimization

**Fig. 5:**
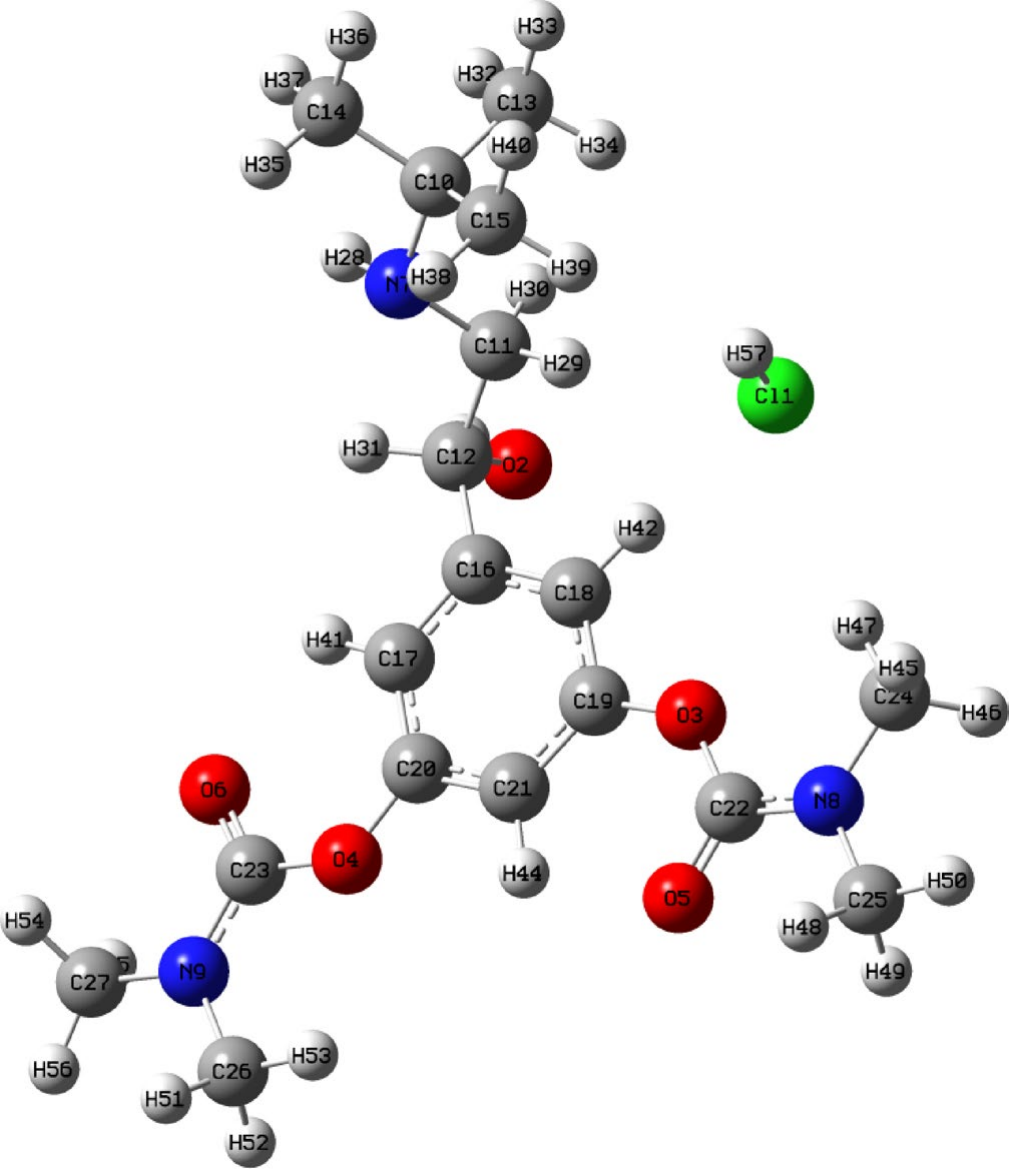
Optimized structures with numbering of atoms of BMBH.

The geometrically optimized parameters of BMBH computed by RB3LYP level with a 6–31 + G (d) basis are listed in Table 3. The corresponding structure and the labelling of atoms, is shown in Fig. 5. The overall geometry has a non-planar structure. The benzene ring’s geometry is perturbed due to different substituents on the benzene ring. The symmetry of the ring is distorted, yielding ring angles greater than 120 at the points of substitution.

**Table 3:** Selected geometrical parameters of BMBH obtained by RB3LYP /6–31 + G(d) levels of DFT.

Bond lengths (A°)	Bond angles (°)	Dihedral angles (°)
O2–C18 1.39	O4–C21–N7 125.8	C21 O3 C22 O5 4.67
O4–C21 1.21	O2–C21–O4 123.39	C15C17 C18 O2 − 176.8
O3–C22 1.37	O3–C19–C20 116.3	C22O3 C19 C20 70.49
N7–C21 1.36	O2–C17–C18 116.8	C18 C20 C19 O3 − 176.19
N7–C23 1.46	C18–O2 –C21 118.5	C22 O3 C19 C15 52.55
N8–C22 1.36	C19–O3–C22 118.95	C25 N8 C22 O3 − 173.99
N6–C10 1.49	C21–N7–C24 124.17	C15C11C10 N6 − 165.11
Cl57– H42 1.87	C22–N7–C25 118.6	
Cl57–H32 3.19	O2–C20–C18 121.20	
	O5–C22–N8 125.6	
	C22––N8–C26 124.2	

#### Mulliken and NBO analyses

Both Mulliken and NBO analyses help predict and rationalize experimental trends in spectroscopy and electrochemistry. They serve as preparatory tools for guiding experiments by predicting molecular properties, and validate experimental data by offering electronic structure explanations. The charge distributions of the molecule have been computed by performing Mulliken analysis. The theoretical calculations of atomic charges play an important role in the application of quantum mechanical calculations to molecular systems. The calculated results have revealed the biggest values of the negative charge at O5, Cl57, and C9. The carbon atoms of the methyl groups are positively charged. Almost very similar values of positive charges are noticed for all hydrogen atoms, forming CH_3_ groups. The highest value of the positive charge is located on H56, connected to N6 (N6-H56).

Natural bond orbital analysis of BMBH is done with NBO 3.1 in Gaussian 09 to confirm charge transfer and conjugation. A second-order perturbation approach, the Fock matrix gives an examination of the energetic importance of electron transfer from the donor (Lewis-type NBOs) to the acceptor (non-Lewis NBOs). The stabilization.

energy, E_(2)_, represents the degree of electron transfer from donor to acceptor and is known as the degree of electron delocalization^38^.

1 $${\text{E}}_{{({2})}} = \Delta {\text{E}}_{{\text{i j}}} = {\text{ q}}_{{\text{i j}}} = \frac{{{\text{F }}\left( {{\text{i}},{\text{ j }}} \right)^{2} }}{{{\text{Ej}} - {\text{Ei}}}}$$

where q _ij_ is donor orbital occupancy, F (i, j) is the off-diagonal Fock matrix element, and E_j_ and E_i_ are the diagonal elements (orbital energies). The E^(2)^ value can represent (reflect) the intensity of the electron donor and electron acceptor and the degree of conjugation of the structure. Table 4 displays the predicted intensities between the donor and acceptor NBOs, from which we can observe the interaction orbitals and their interaction intensities. Intramolecular interactions arise from π(C–C) → π*(C–C) overlap and LP → σ*(O-C) and (N–C) transfer. These contribute to charge transfer and hydrogen bonding, making the main contribution to the stabilization of the system.

**Table 4:** Second-order perturbation analysis of the Fock matrix of BMBH.

F (i,j) a.u	Ej-Ei a.u	E ^(2)^ kcal/mol	Acceptor j	Donor i	
0.059	0.37	10.71	${\pi }^{*}$ C16-C19	LP (^2^)O3	(1)
0.130	0.58	35.92	${\sigma }^{*}$ O2-C21	LP (^2^)O4	(2)
0.117	0.72	22.60	${\sigma }^{*}$ N7- C21	LP (^2^)O4	(3)
0.130	0.58	35.62	${\sigma }^{*}$ O3- C22	LP (^2^)O5	(4)
0.117	0.72	22.60	${\sigma }^{*}$ N8- C22	LP (^2^)O5	(5)
0.113	0.35	45.57	${\sigma }^{*}$ O4- C21	LP (^1^)N7	(6)
0.112	0.36	43.99	${\sigma }^{*}$ O5- C22	LP (^1^)N8	(7)
0.190	0.36	102.55	LP* H27	LP (^4^) Cl57	(8)
0.082	0.01	220.31	C15-C17	BD (^2^) *C18-C20	(9)
0.082	0.01	231.09	C15-C17	BD (^2^) *C16-C19	(10)

A significant contribution to the stabilization energy could be added by LP Cl57 ⟶ LP^*^ H27 at a value of 102.55 in E ^(2)^ (Table 4).

#### Frontier molecular orbitals (FMOs)

**Fig. 6:**
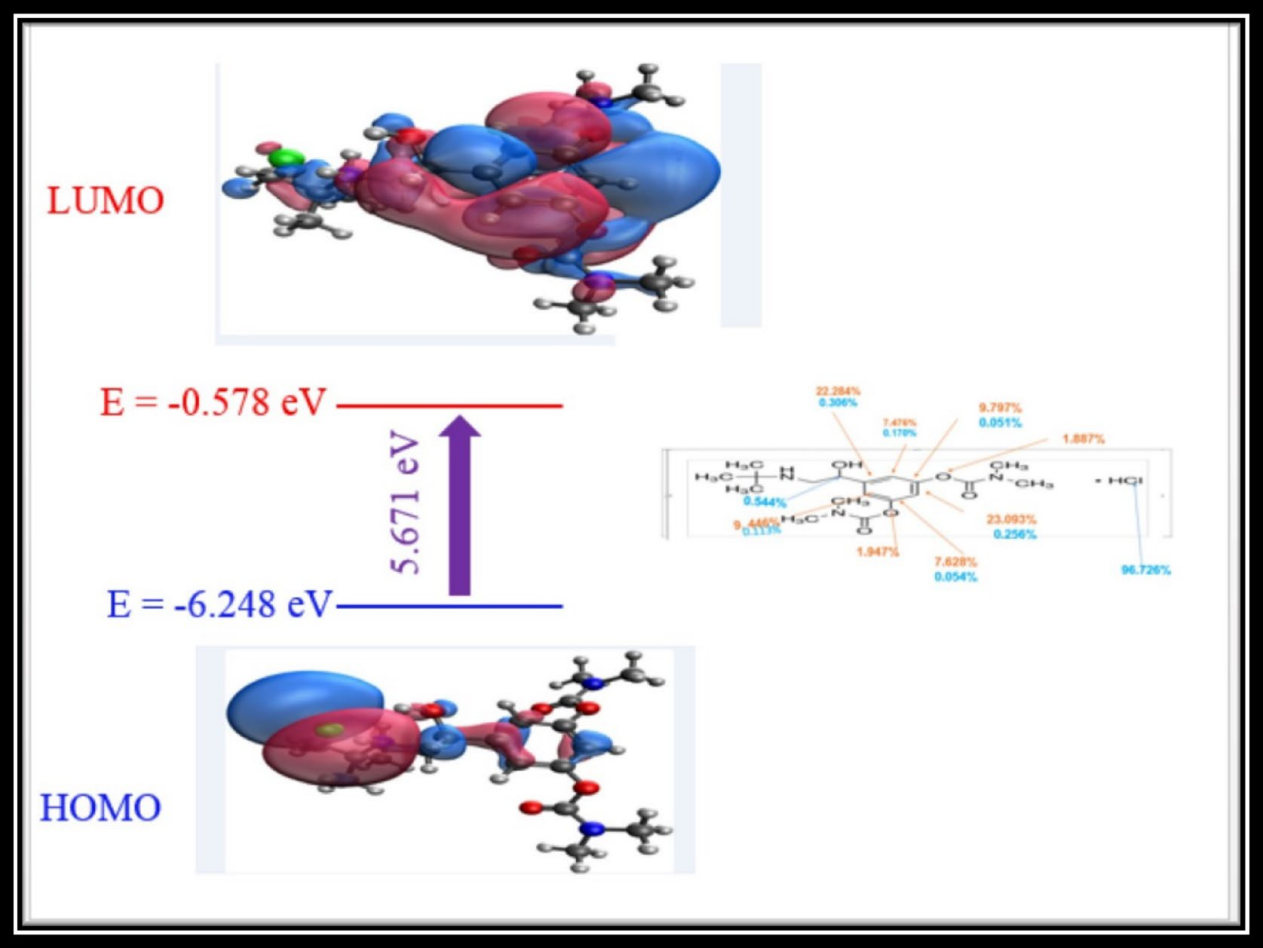
The atomic orbital compositions of the frontier molecular orbitals of BMBH were calculated by the Becke method. Percentage contributions to the HOMO and LUMO are given in blue and red colors respectively.

**Fig. 7:**
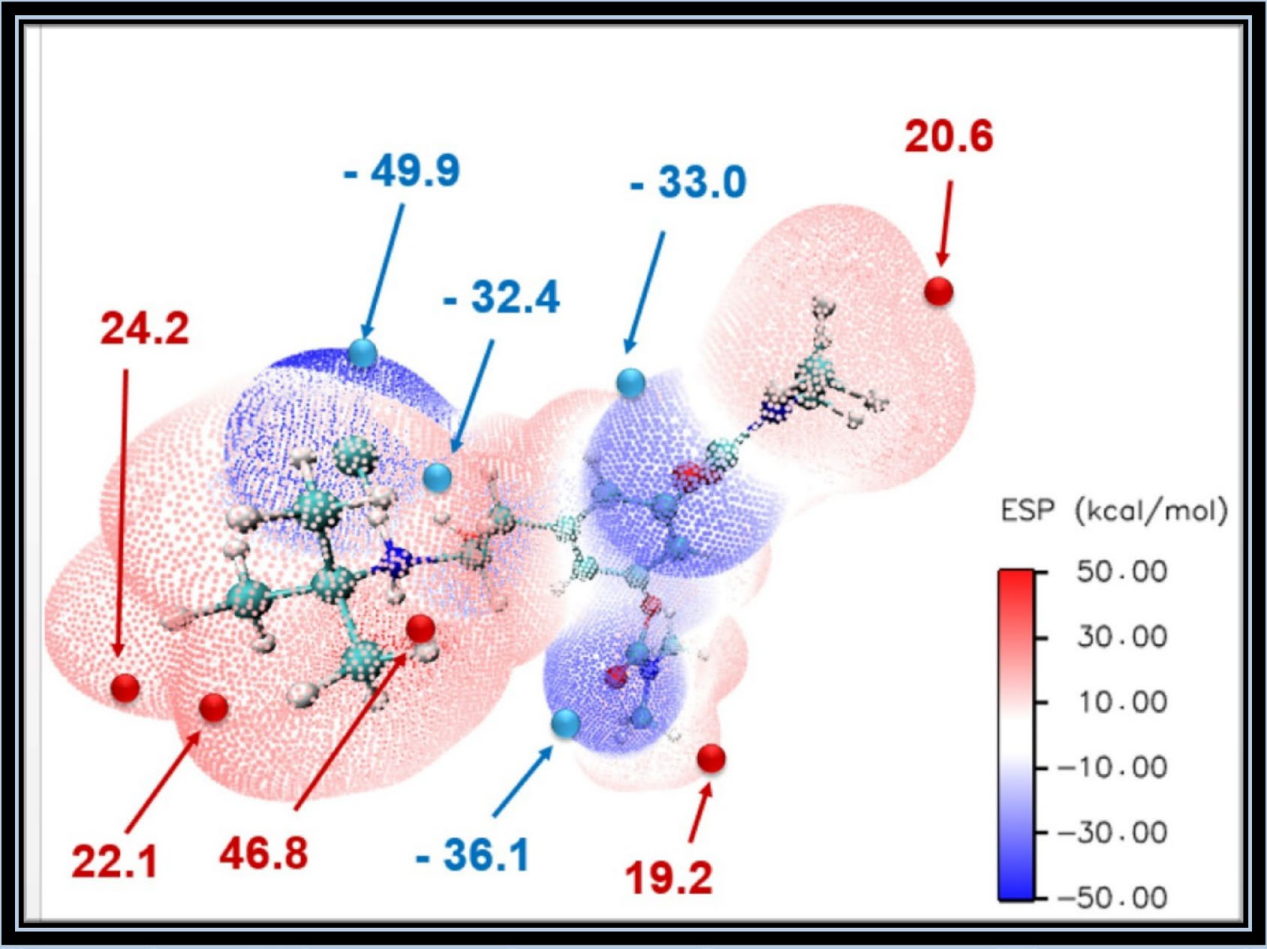
3D molecular electrostatic potential map for BMBH along with the values of its electrostatic and vdW surfaces with colored scale potential values.

Frontier Molecular Orbital analysis is a powerful theoretical tool that enhances the interpretation of experimental data in various fields of chemistry, materials science, and biochemistry. The highest occupied molecular orbital (HOMO) and the lowest unoccupied molecular orbital (LUMO)energies, along with their gap, provide fundamental insights into electronic transitions, chemical reactivity, and molecular stability. HOMO and LUMO energies of the title compound were computed with the same level of DFT theory and are shown pictorially in Fig. 6. The compositions of both HOMO and LUMO were calculated by the Becke method via the Multiwfn program. From Fig. 7, it can be seen that the HOMO result from the lone pair present on the chloride atom contributes to the HOMO by 96.7%. The LUMO results mainly from the aromatic system (benzene ring), with a contribution of 78%. The chemical reactivity of the title compound would be assessed based on the global reactivity descriptors. The energy of the HOMO and LUMO is directly related to the ionization potential (IP) and electron affinity (EA).

The value of IP and EA are given according to Koopman^’^s theorem^39^.

2 $${\text{IP}} = - {\text{HOMO}}$$

3 $${\text{EA }} = - {\text{LUMO}}$$

The values of IP and EA can be used to deduce the global reactivity descriptors, including chemical potential (µ), chemical hardness (η), chemical softness(S), electronegativity (X), and electrophilic index (ω) according to the following equations.

4 $$\upmu \, = - \left[ {\left( {{\text{IP}} + {\text{EA}}} \right)/{2}} \right]$$

5 $$\upeta = \, \left( {{\text{IP}} - {\text{EA}}} \right)/{2}$$

6 $${\text{S }} = { 1}/{2}\upeta$$

7 $${\text{X }} = \, \left( {{\text{IP }} + {\text{ EA}}} \right)/{2}$$

8 $$\upomega \, = \, \upmu^{{2}} /{2}\upeta$$

According to the values in Table 5, (E)/ (Nmax) is − 1.707eV, and the maximal charge acceptance Nmax is 1.203eV. These values reveal the drug’s intramolecular charge transfer as well as its ability to interact with and bind to β1-adrenergic receptors.

**Table 5:** Activity parameters of BMBH, including key chemical reactivity descriptors.

Chemical reactivity descriptor	Value
Ionization potential (*I*)	6.248 eV
Electron affinity (*A*)	0.578 eV
Electronegativity (χ)	3.414 eV
Chemical potential (*µ*)	− 3.414V
Chemical hardness (η)	2.836 eV
Chemical softness (*S*)	0.352 eV
Electrophilicity index (ω)	2.055 eV
Energy change (Δ*E*)	− 2.055 eV
Maximal charge acceptance (Δ*N*_*ma*_)	1.203 eV
ΔE/Δ_max_	− 1.707 eV

The chemical hardness (η) and the electron transfer energy (E) are 2.836 and -2.055 eV, respectively. These findings suggest that the charge transfer process in the drug and bioactivity formation of intermolecular interaction with 1-adrenergic receptors and blocking is permissible^40^. Relative to biological impact, a higher HOMO–LUMO gap (5.671 eV) and higher ω (2.055 eV) predicted the interaction of the designed structure with nucleophilic sites in DNA (e.g., guanine N7) or protein functional groups (–SH, –NH₂). Additionally, BMBH has more negative HOMO energy (− 6.248 eV), it may act as an electron acceptor, potentially forming hydrogen bonds or covalent interactions with biomolecules. These properties can influence binding affinity, biodegradation, and toxicity, which are important for drug design or toxicity assessment^41,42^.

#### The molecular electrostatic potential (MEP) map

To predict reactivity, MEP identifies nucleophilic and electrophilic sites, also it analyzes biological recognition and hydrogen bonding. One can use the concept of molecular electrostatic potential (MEP), which is connected to electron density^43^. Drug-receptor interactions and the electrostatic potential (ESP) V(r) have both been extensively studied^44,45^. The 3D ESP map shows potential values at electrophilic and nucleophilic sites. The most electrostatically positive, most negatively charged, and zero electrostatic potential regions are represented by the colors blue, red, and white on the MEP surface, respectively. Figure 7 shows surface maxima for positive potential sites surrounding the hydrogen atoms and 4 surface minima for the negative potential sites on chlorine and oxygen atoms. The global minimum on the surface (− 49.9 kcal/mol) is located on Cl57. While the location of the surface’s global maxima (+ 46.8 kcal/mol) is located on H27, we note that the strongest nucleophilic and electrophilic sites, as well as the global maximum and minimum, are found in the vicinity of hydrogen chloride. The MEP map shows that the hydrogen chloride region is highly biologically active and crucial for drug recognition.

#### Density of states (TDOS and PDOS)

TDOS and PDOS are essential computational tools that help explain and predict experimental results across spectroscopy, solid-state physics, and electrochemistry. Their theoretical insights bridge the gap between quantum calculations and real-world materials behavior, aiding in material design and technological advancements.

The Partial Density of States (PDOS) of BMBH is a crucial property in computational material science and quantum chemistry. It describes the contribution of specific atomic orbitals or elements to the total electronic Density of States (DOS). It provides insights into the molecule’s electronic structure, bonding characteristics, and reactivity^46^.

**Fig. 8:**
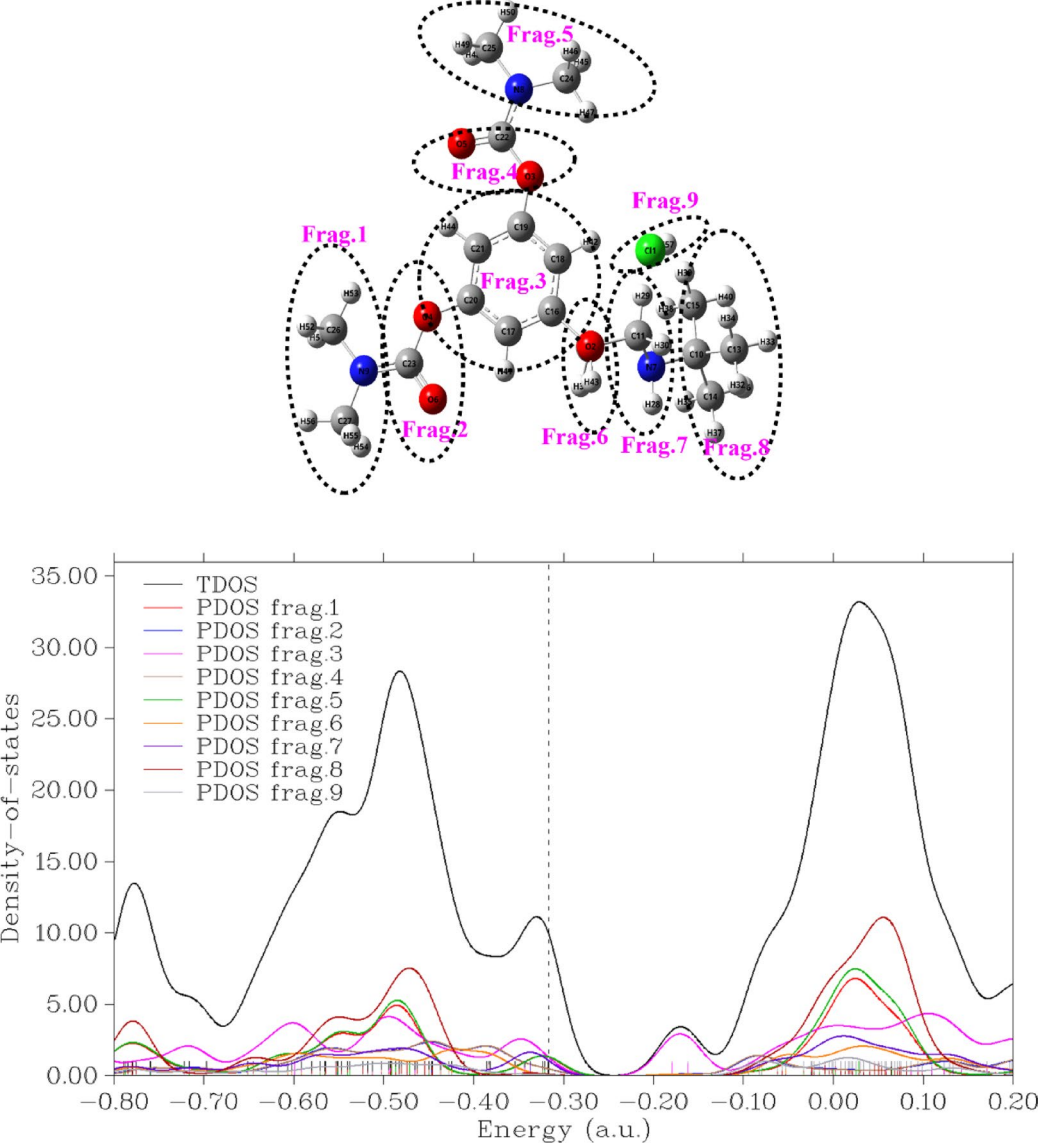
Total density of states (TDOS) and partial density of states (PDOS) for fragments of BMBH, illustrating the electronic structure and contribution of different molecular orbitals.

BMBH, a prodrug of terbutaline, consists of a complex molecular structure containing elements such as carbon (C), hydrogen (H), oxygen (O), nitrogen (N), and chlorine (Cl). By calculating the PDOS using Density Functional Theory (DFT) methods, we can analyze how different atomic orbitals contribute to the electronic states near the Fermi level. The structure was classified into fragments, where each part could export the main contributed levels to form the structure identity. From Fig. 8, it was observed that Fragments 8, 5, and 1 can contribute the most contribution of the electronic levels in leading to the structure stability. However, fragment 9 (HCl) shares with minimum orbital contribution, concluding its interaction with a weak H-bonding nature inside the molecule.

#### Reduced density gradient ِ (RDG) analysis

RDG provides a deeper understanding of noncovalent interactions, guiding the interpretation of experimental results and assisting in the design of new materials, drugs, and catalysts. Equation 9 gives the reduced density gradient (RDG), which is a fundamental dimensionless quantity derived from the density and its first derivative^47^.

9 $${\text{RDG(}}r{)} = \frac{1}{{2\left( {3\pi^{2} } \right)^{\frac{1}{3}} }}\frac{\nabla \rho (r)}{{\rho (r)^{\frac{4}{3}} }}$$

Large negative values of sign (λ_2_) in RDG tails indicate attractive interactions (such as dipole–dipole or hydrogen bonding); if the sign (λ_2_) is large and positive, the interaction is non-bonding (steric effect).

**Fig. 9:**
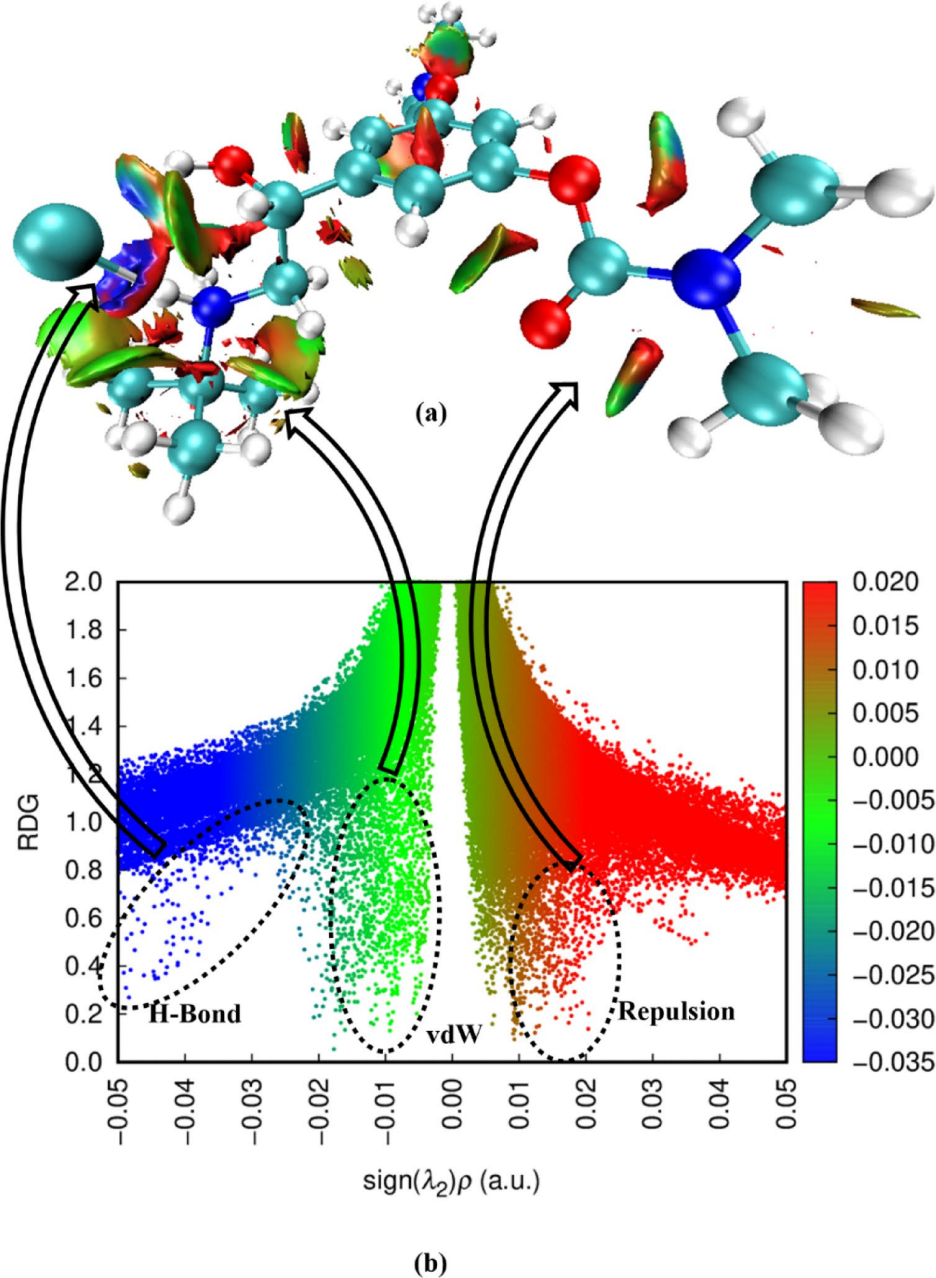
(**a**) RDG map versus the electron density (ρ) multiplied by the sign λ_2_ for BMBH (**b**) The surfaces are colored on a blue-green–red scale according to values of sign λ_2_.

In Fig. 9, green colors are identified as van der Waals interactions, while the red color is identified as strong repulsion. Repulsive interactions were observed inside the ring, while van der Waals interactions were observed between the hydrogen atoms. The negative value of the sign (λ_2_ ) shows the weak hydrogen bonding interactions^48,49^.

#### Electron localization function (ELF)

The Electron Localization Function (ELF) serves as a valuable tool for analyzing the electronic structure, bonding nature, and atomic shell distribution across the molecular surface. The electron distribution within the compound is analyzed and interpreted using the Electron Localization Function (ELF) and Localized Orbital Locator (LOL). The fundamental principle governing ELF is Pauli repulsion, which arises due to an excess of kinetic energy density. Regions where opposite spin-paired electrons or single electrons exhibit maximal localization are characterized by an ELF value approaching 1, indicating areas of maximum Pauli repulsion. Conversely, regions with minimal electron localization correspond to ELF values near 0. Due to Pauli exclusion forces, highly localized electron regions delineate atomic shells, chemical bonds, and lone pair electrons, effectively explaining the most repelled electronic domains^50^.

**Fig. 10:**
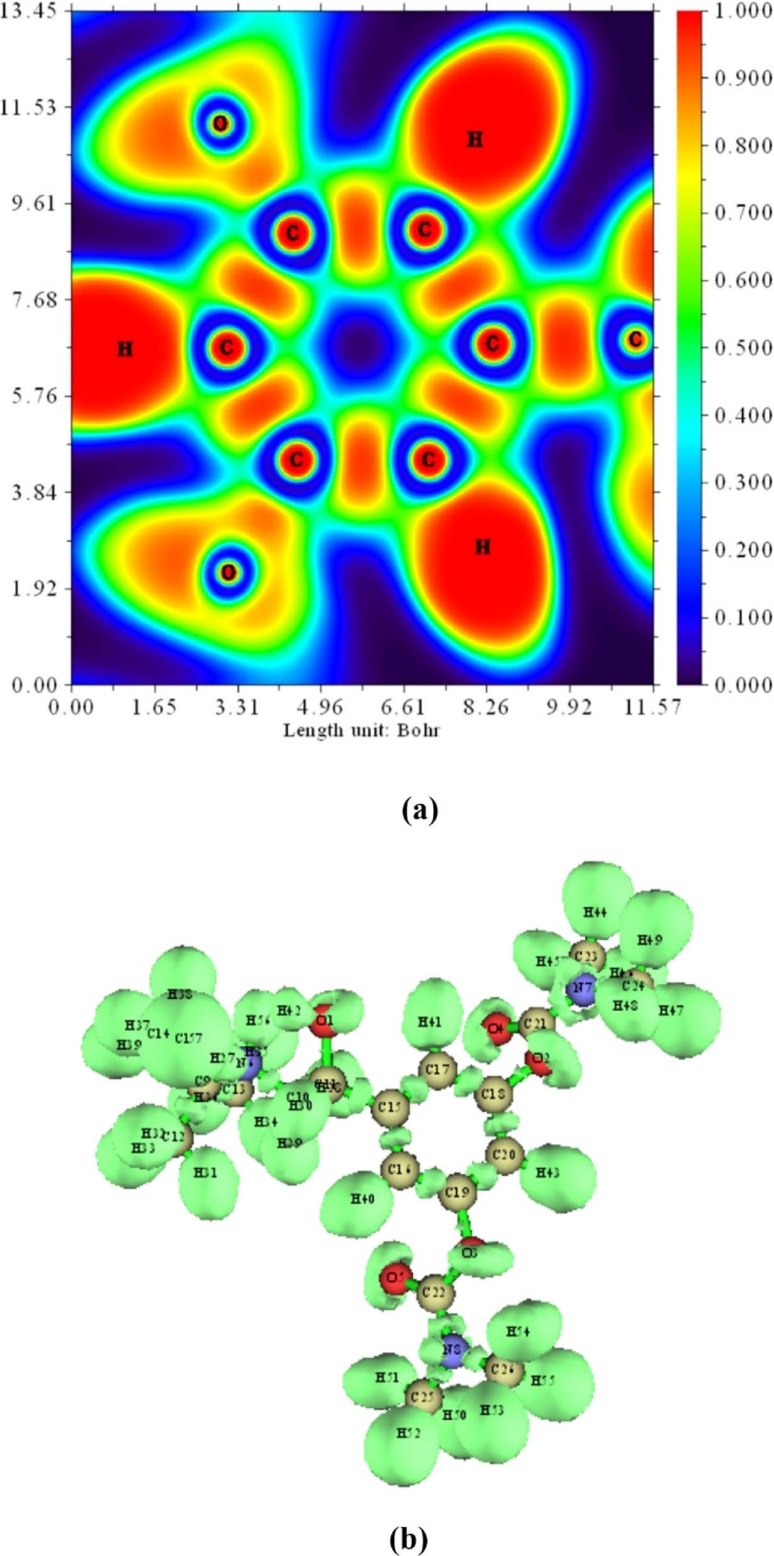
(**a**) Electron localization function (ELF) color map, and (**b**) 3D plots ELF-isosurfaces, for BMBH with depicting the electron density distribution and localization in the molecular structure.

The ELF contour map, generated using Multiwfn software and displayed in Fig. 10a, highlights regions of varying electron density. High ELF values (0.85–1.0 Bohr) signify strong electron localization, indicative of covalent bonding, whereas low ELF values (0.0–0.4 Bohr) reflect pronounced electron delocalization. The ELF visualization further reveals intense electron concentration around hydrogen atoms, depicted in red, while delocalized electron regions surrounding oxygen and carbon atoms appear in blue. Notably, strong electron localization is observed between carbon atoms within the ring and bonded hydrogen atoms, resulting from the overlap between the carbon sp orbitals and hydrogen s orbitals. Additionally, the perturbation in ELF distribution near oxygen atoms is attributed to interactions involving hydrogen and halogen bonds.

The three-dimensional (3D) ELF plot for BMBH is presented in Fig. 10b. In this representation, monosynaptic basins correspond to lone electron pairs, whereas disynaptic basins are indicative of covalent interactions. Monosynaptic regions of oxygen, nitrogen, and chlorine are widely distributed. Disynaptic basins of OH, NH, and CH bonds are more confined due to hydrogen and halogen bonding.

#### AIM calculations

**Fig. 11:**
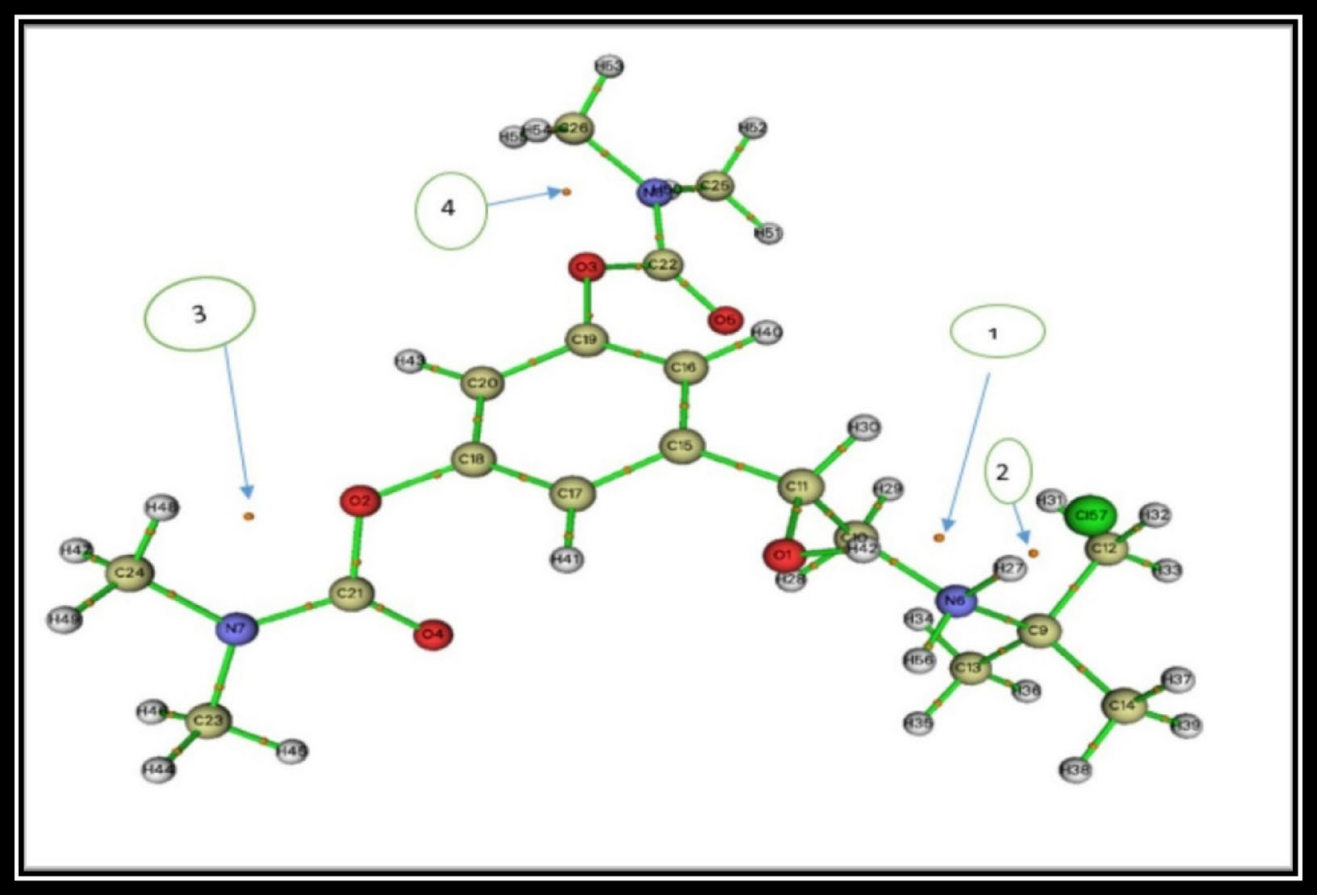
Bond critical points (BCP) for BMBH using AIM analysis.

The atoms in molecules (AIM) theory are a convenient method to analyze the hydrogen bonding and other interactions in various molecular systems. It has been extensively used to classify and understand bonding interactions in terms of quantum mechanical parameters and their derivatives as electron density (*ρ*). The theory of AIM has efficiently described the H-bonding, and it^'^s a concept without borders. One of the advantages of this theory is that one can obtain information on the change in electron density distribution as a result of their bond formation or complex formation^51^. The molecular graph of the molecule using AIM theory is shown in Fig. 11. The topological parameters of non-covalent interactions are grouped in Table 6. The AIM results show that BMBH is characterized by 4BCPs of non-covalent character. Two describe hydrogen bonding, and two characters halogen bonding interactions. According to the values of the parameters reported in Table 5. We can classify the non-covalent interactions as weak hydrogen and halogen bonding interactions, except for N6-H27……Cl57 strong bonding interactions^52^ (Table 6; Fig. 11).

**Table 6:** Topological parameters of the non-covalent interactions of BMBH.

E_bond_ kJ/mol	ELF (a.u)	V ( r ) a.u Localization potential	${\nabla }^{2}$ *ρ*(r) a.u Laplacian of the electron density	*ρ*(r) a.u Electron density	Interactions
− 19.7	0.08778	− 0.01506	0.061778	0.001682	O1–H42…CL57
− 57.95	0.30722	− 0.044249	0.10121	− 0.009472	N6–H27…CL57
− 18.73	0.03816	− 0.014301	0.08193	0.003090	C21–O2 …H48
− 18.36	0.038257	− 0.01402	0.082101	0.003095	C26–H54….O3

#### Nonlinear optical (NLO) effects

The nonlinear optical (NLO) effects arise from the interaction of electromagnetic fields. It is applied in various media to produce new fields altered in phase, frequency, amplitude, optical switching, optical logic, and other propagation characteristics from the incident fields. The molecules with NLO properties have been extensively investigated due to their applications in the areas of signal processing, telecommunication, and optical interconnection. The calculations of the total molecular dipole moment (µ), mean linear polarizability (α), and mean first hyperpolarizability (β) from the Gaussian output have been explained in detail previously^53^. These parameters help in optimizing the molecular modifications of the studied structure, aiming to improve the pharmacological effects.

Table 7 shows the dipole moment, polarizability, and first hyperpolarizability components of the drug compound, whose invariant is calculated with a numerical derivative of the dipole moment using the RB3LYP/6–31 + G(d) level of DFT. The total static dipole moment, average linear polarizability, anisotropy of polarizability, and first hyperpolarizability were calculated according to the reference^54^.

**Table 7:** Calculated dipolemoment µ (Debye), polarizability(α)and thefirst hyperpolarizability (β) components(a.u.) of BMBH.

Values	Components	Values	Components
246.283	*β*_*xxx*_	5.81840	*µ*_*x*_
− 303.285	*β*_*xyy*_	− 1.48979	*µ*_*y*_
− 3.73170	*β*_*xzz*_	− 6.12364	_*z*_ *µ*
− 146.087	*β*_*yyy*_	308.136	*α *_*xx*_
− 26.1894	*β*_*xxy*_	307.742	*α *_*yy*_
− 142.729	*β*_*yzz*_	222.910	*α*_*zz*_
− 62.19	*β*_*zzz*_	4.89543	*α *_*xy*_
28.1994	*β*_*xxz*_	1.15448	*α *_*xz*_

The calculated values of total static dipole moment µ, the average linear polarizability α, the anisotropy of the polarizability ∆α, and the first hyperpolarizability β using the RB3LYP/6–31 + G(d) level of the DFT method are 8.5 Debye, 297.5 a.u., 540.42 a.u., and 2.676 × 10^−30^ e.s.u.

Urea is one of the prototypical molecules used in the study of the NLO properties of molecular systems, and it is frequently used as a threshold value for comparative purposes. The values of µ, α, and β obtained with the RB3LYP/6–31 + G(d) method for urea is 1.373 Debye, 3.831 Å3, and 3.729 × 10^−31^ cm^5^ e.s.u.^−1^, respectively^55^. The drug compound’s first- and second-order polarizabilities are greater than those of the urea. The title compound may be a potential candidate in the development of NLO materials based on the magnitude of its first hyperpolarizability. As a result, this molecule could serve as a potential building block for nonlinear optical materials.

### Simulation of molecular dynamics for BMBH interacting with a graphene surface

**Fig. 12:**
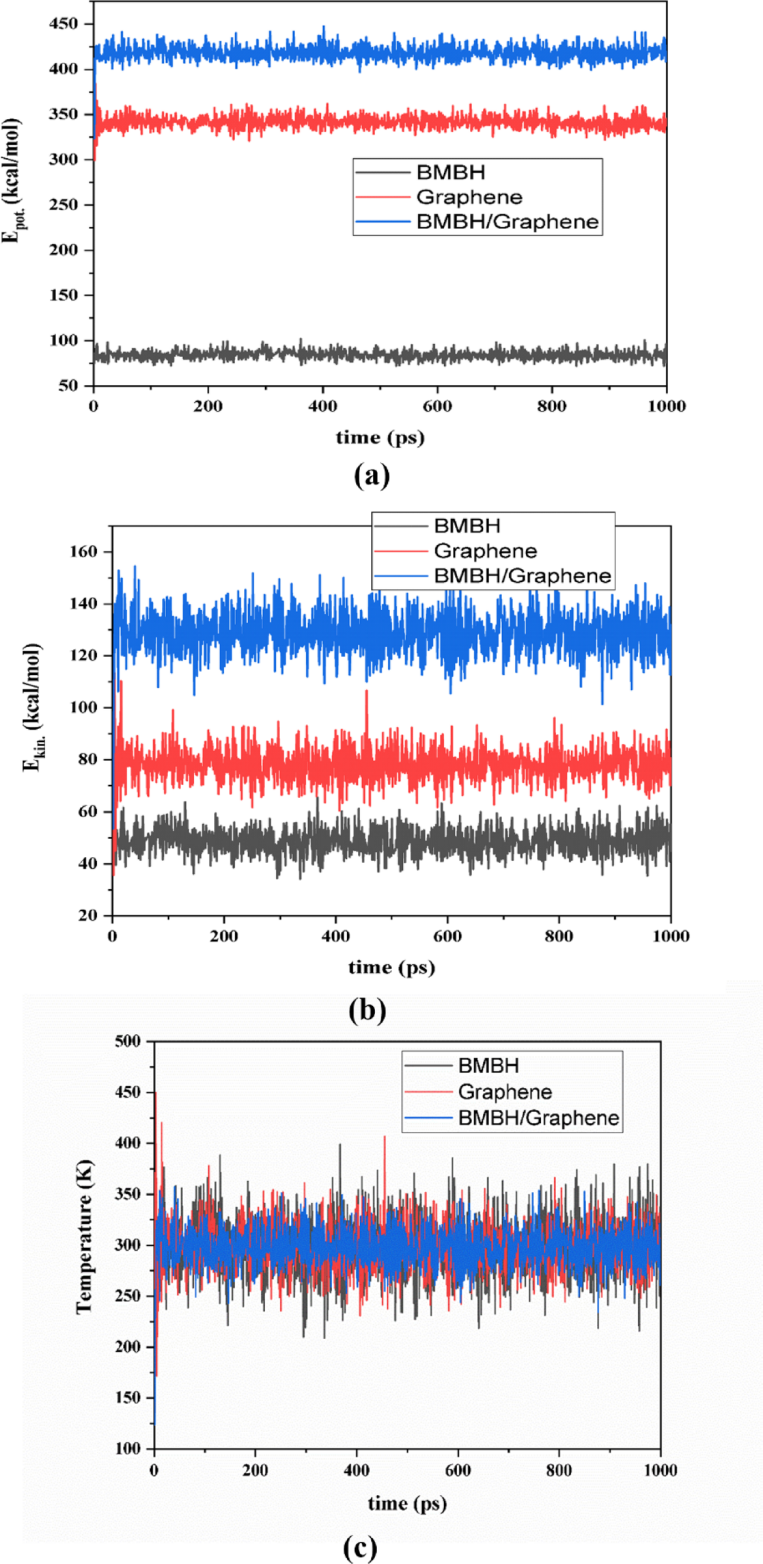
(**a**) potential energy variable, (**b**) kinetic energy variable for the designed systems before and after adsorption during dynamic simulation, and (**c**) temperature change through simulation time for un adsorbed and adsorbed systems.

MD simulation in solid state adsorption systems can support the stability of the designed models and increase the prediction of drug transport in pharmaceutical approaches. The designed BMBH/Graphene system was studied via the noncovalent interaction computational method GGA/PBE related to the DFT category. The Forcite module was utilized with ensemble NVT to describe the energy and temperature changes through the simulation time of 1000 ps. The dynamic analysis estimates values of potential energy (E_pot_) and kinetic energy (E_kin_) for each simulation step before and after adsorption. Figure 12a,b illustrate the energy parameter changes for the separated BMBH and graphene system compared with their adsorption system. As shown, the energetic variables are low with BMBH drug, while the other species exported larger energetic simulations with both E_pot_ and E_kin_. However, the potential energy curves in the exported significantly higher values with the BMBH/graphene adsorption system. Figure 12c shows the changes in temperature for each system, and this shows less flocculated values with the BMBH/graphene adsorption system, while all designed curves dynamically moved around 300 K. The results predicted some stability in the adsorption formed system, which may be motivated by the drug delivery approach.

### BMBH/graphene adsorption energy estimation

**Fig. 13:**
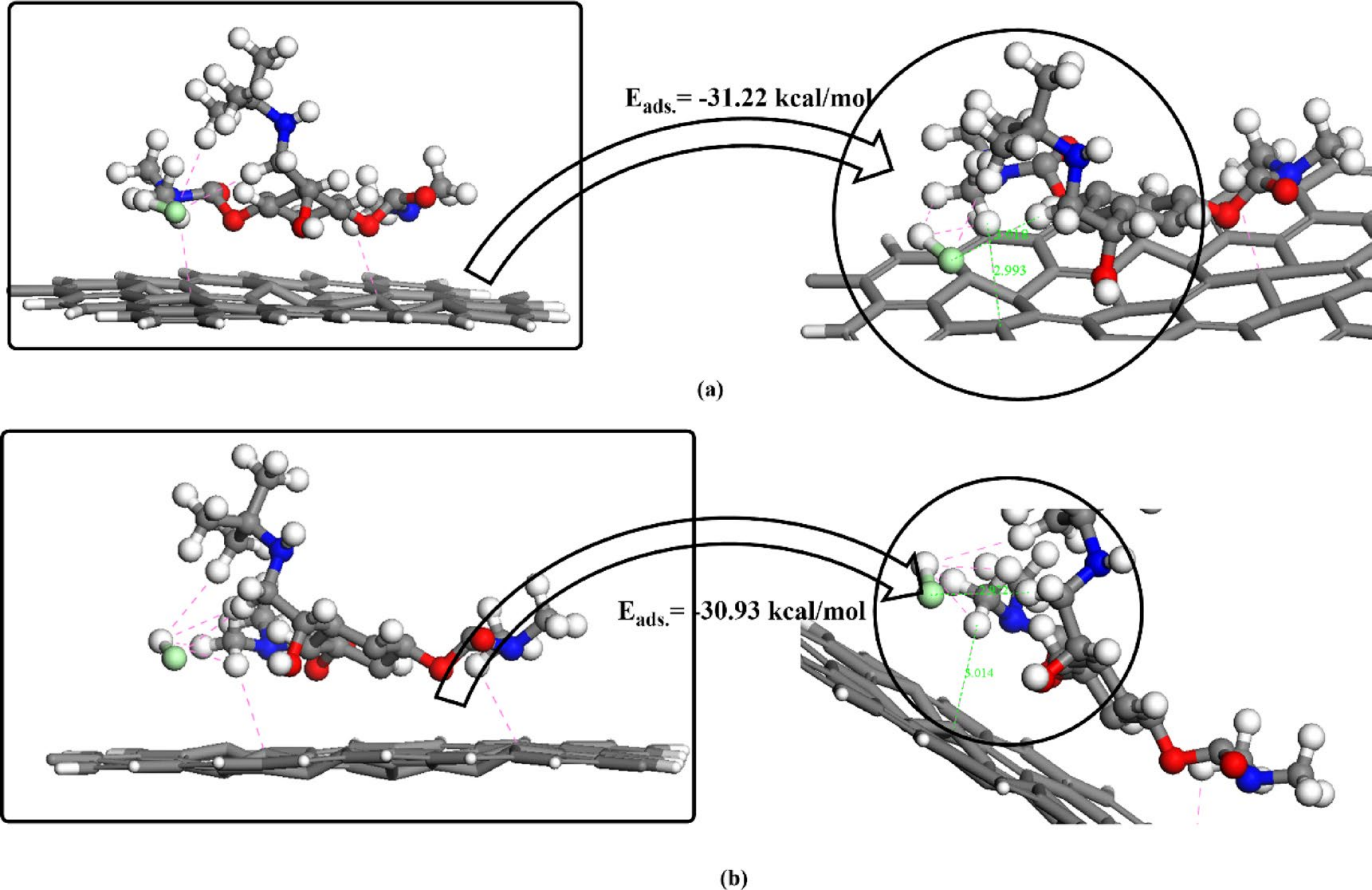
The best BMBH/graphene adsorption models using the adsorption locator module.

**Fig. 14:**
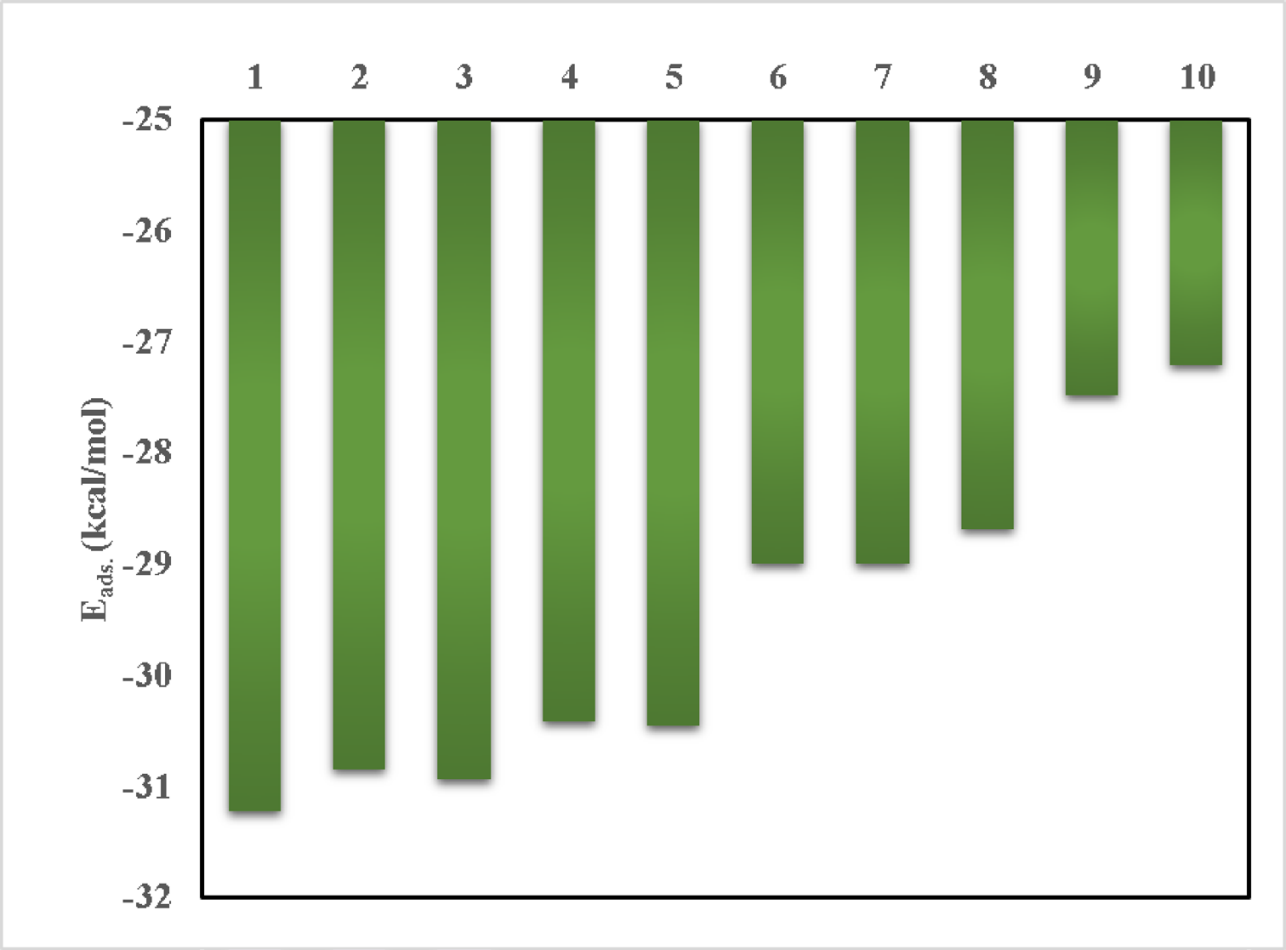
Correlation between the adsorption energy parameter and the interaction system type across 10 generated models for BMBH/graphene adsorption system.

To predict the noncovalent interactions, present on the surface of Graphene via the adsorption of BMBH, GGA/PBE geometric optimization was followed by an examination of the adsorption location. The adsorption locator module predicted the preferred adsorption mechanistic models, in which the first model was arranged to be more stable, to confirm the occurrence of some interactions in the adsorption mechanism. The difference between a species’ energies before and after adsorption can be used to anticipate the system’s calculated adsorption energy^56–58^.

10 $$E_{ads} = E_{{{\text{BMBH}}/{\text{Graphene}}}} - \, ({\text{E}}_{{{\text{BMBH}}}} + E_{Graphene} )$$

where *E*_BMBH_ is the energy of the adsorbate, *E*_Graphene_ is the energy of the adsorbent surface and *E*_BMBH/Graphene_is the overall energy of the adsorption system. The interaction of BMBH on the graphene surface can be controlled with hydrogen atoms interacting with the π-electronic mobility of the graphene phenyl ring. Also, other intramolecular close contacts between Cl and H of the CH_3_ group can support the system’s stability. This adsorption study may be helpful in several applications such as drug delivery and excess drug removal in medical and pharmaceutical strategies. As illustrated in Fig. 13, two best-proposed interaction models exhibit close-contact non-covalent interactions, which are primarily ranged from 10 designed models for BMBH/Graphene adsorption system. The adsorption behavior is characterized by the energetic parameter values. The adsorption energy values resulting from surface interactions exported the order from 1 to 10 as shown in Fig. 14, where models number 1 and 3 estimated the optimum binding affinity (− 31.22 kcal/mol and − 30.93 kcal/mol, respectively). The predicted adsorption study may give an important insight into system stability aiming to hydrogen storage on the electronic-rich surface. The noncovalent interactions present on the surface can be summarized as the adsorption stability of BMBH on Graphene is governed by π-π stacking, electrostatic interactions, hydrogen bonding, and charge transfer effects. The moderate binding energy suggests its potential for reversible adsorption, making it useful for drug delivery and hydrogen storage applications.

**Table:** 

Structures	Total energy	Adsorption energy	Rigid adsorption energy	Deformation energy	compound : dEad/dNi
3D Atomistic-1	4.764215	− 198.539	− 31.2231	− 167.315	− 198.539
3D Atomistic-2	5.027971	− 198.275	− 30.8372	− 167.438	− 198.275
3D Atomistic-3	5.682667	− 197.62	− 30.9342	− 166.686	− 197.62
3D Atomistic-4	5.916225	− 197.386	− 30.4179	− 166.969	− 197.386
3D Atomistic-5	6.542835	− 196.76	− 30.4465	− 166.313	− 196.76
3D Atomistic-6	6.795467	− 196.507	− 28.9929	− 167.514	− 196.507
3D Atomistic-7	7.077594	− 196.225	− 28.9945	− 167.231	− 196.225
3D Atomistic-8	7.609889	− 195.693	− 28.6793	− 167.014	− 195.693
3D Atomistic-9	7.97483	− 195.328	− 27.4851	− 167.843	− 195.328
3D Atomistic-10	8.193138	− 195.11	− 27.2049	− 167.905	− 195.11

### Molecular docking analysis

**Fig. 15:**
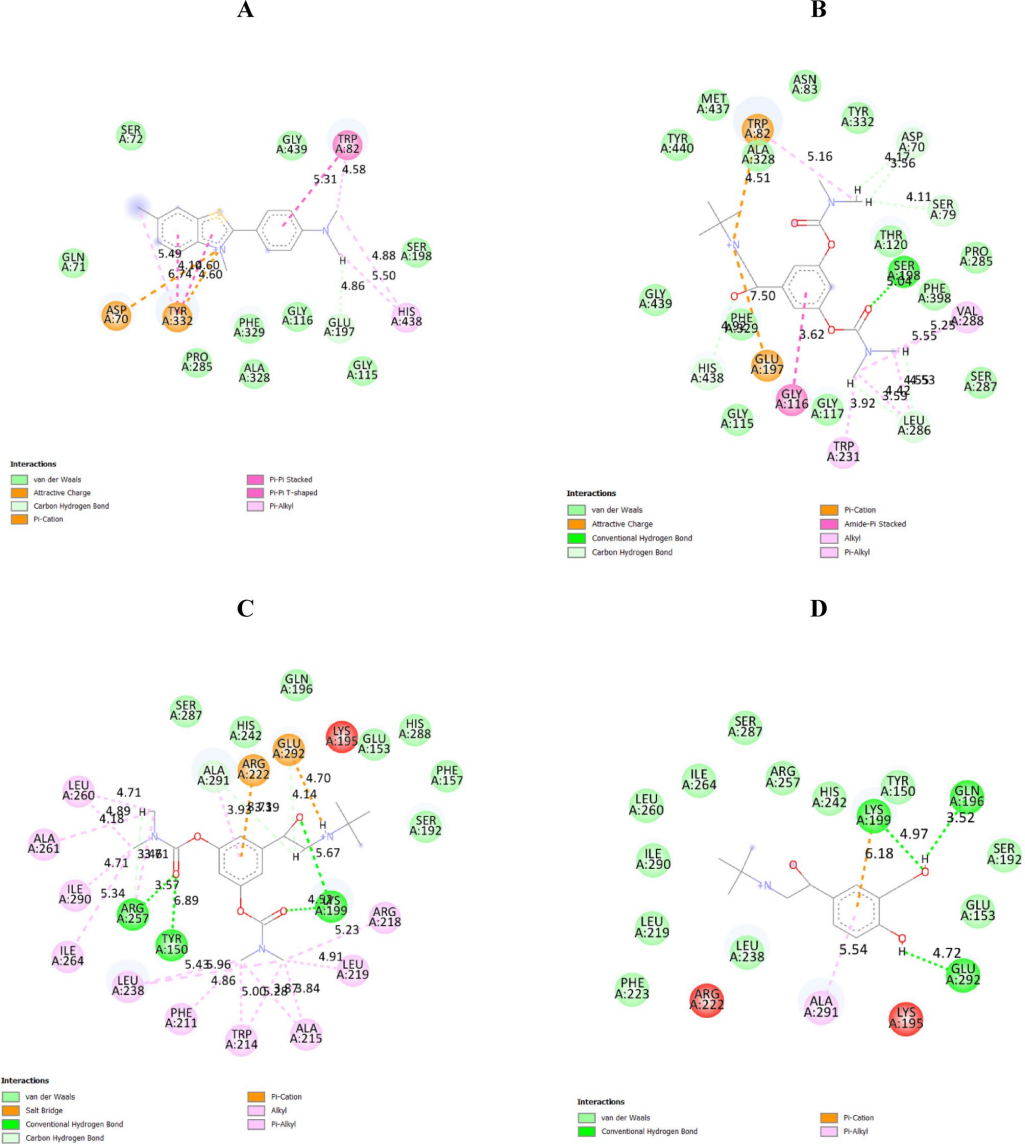
Molecular docking analysis of thioflavine T against BChE (**A**), BMBH against BChE (**B**), BMBH against HSA (**C**), and salbutamol against HSA protein (**D**).

Docking protocol was validated by re-docking of the co-crystalized thioflavine T at the active site of butyrylcholinesterase (PDB ID: 6ESY), Fig. 15A. the re-docking rmsd = 1.16 Å and binding score =  − 5.75 kcal/mol. All the key interactions accomplished by the co-crystalized ligand with the key amino acids in the binding site are reproducible using the docking setup, mentioned in the experimental section. The validated docking setup was then used to investigate the ligand-receptor interactions and binding patterns for bambuterol hydrochloride (score =  − 7.99 kcal/mol), Fig. 15B. The amino acid residues involved in interaction at binding site with co-crystallized thioflavine T are.

Asp70, Trp82, Tyr332, where the main interactions are ionic and π-cation with amino acid residues Asp70 and Tyr332 and π–π stacking with Trp82.

The docking investigation showed that bambuterol hydrochloride depicted higher binding interactions than thioflavin T. Still, the common residues like Trp82 which was able to form ionic and π-cation interactions. Additionally, ionic interaction was observed between BMB oxygen atom and Ser198, ionic bond with Glu197 and BMB ammonium nitrogen and amide- π interaction between BMB aromatic ring and Gly116 (Table 8). All of which justify the lower binding score and higher affinity of bambuterol than thioflavin T towards the butyrylcholinesterase enzyme.

**Table 8:** Energy scores (kcal/mol) and interactions for bambuterol hydrochloride, thioflavine T and salbutamol against BChE and HAS.

Receptor	Compound	Energy score (S) (kcal/mol)	Amino acids involved in interaction
BChE	Thioflavine T	− 5.75 rmsd 1.16	Asp70
			Trp82
			Tyr332
	Bambuterol	− 7.99	Trp82
			Gly116
			Glu197
			Ser198
HSA		− 8.32	Lys199
			Tyr150
			Arg257
			Glu292
			Arg222
	Salbutamol	− 6.35	Glu196
			Lys199
			Gln292

These interactions suggest that BMBH has a high affinity for BChE, which may impact its inhibitory potential against the enzyme. This interaction is significant because BChE is involved in the breakdown of acetylcholine, and its inhibition can enhance cholinergic transmission. BMBH’s inhibition of this enzyme could lead to increased acetylcholine levels, which is beneficial for treating neurodegenerative diseases such as Alzheimer’s disease. This suggests that BMBH could be a promising candidate for further development as a cholinesterase inhibitor, potentially improving cognitive function in patients with cholinergic deficits.

Secondly, to evaluate the bioavailabilty of BMBH, it was docked against Human Serum Albumin (HSA), a highly abundant multifunctional protein in the blood plasma, and compared to a well-known β₂-adrenergic agonist (salbutamol). HSA plays a critical role in transporting a wide range of endogenous and exogenous molecules, including drugs, metabolites, and fatty acids. Docking against HSA is important as many natural compounds, such as plant-derived bioactives, bind to HSA. Docking studies can help assess whether a compound can efficiently utilize this transport mechanism to reach its target tissues. The interaction of BMBH with the HSA binding site results in a high binding energy of -8.32 kcal/mol, which provides insight into the compound’s high binding affinity. Three principal interactions are observed in Fig. 15C: 3 hydrogen bonds with Lys199, Tyr150, and Arg257, the salt bridge with Glu292 and Pi-cation interaction between the ligand’s aromatic ring with Arg222. Furthermore, hydrophobic interactions contributed to the ligand’s high binding affinity, with hydrophobic residues observed to be involved in this interaction. On the other hand, less binding affinity is observed between β₂-adrenergic agonist (salbutamol) and HSA forming a π-cation interaction with Lys199 and 3 H-bonds with Glu196, Lys199, and Gln292 (Fig. 15D).

This difference in the binding affinities against HSA protein indicates that BMB might have a longer duration of action compared to other β₂-agonists while salbutamol exhibits quicker onset but shorter action.

### Molecular dynamics analysis

**Fig. 16:**
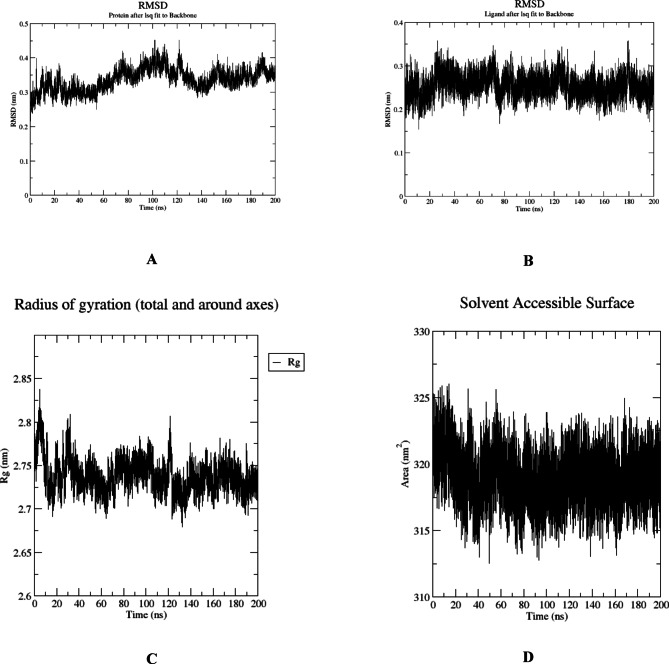
Structural dynamics of HSA RMSD (**A**), BMBH RMSD (**B**), complexes radius of gyration (**C**), and SASA (**D**) calculated during the 200 ns of MD trajectories.

The results presented highlight the dynamic behavior of the HSA protein upon binding with BMBH, as well as the behavior of the ligand itself throughout the 100 ns simulation trajectory. Firstly, focusing on the RMSD of the HSA protein upon BMBH binding, it is evident that there is a stable fluctuation observed over the course of the simulation. The average RMSD value of approximately 0.35 nm indicates that the protein maintains a relatively stable conformation, suggesting that the binding event with BMBH does not induce significant structural changes in the protein (Fig. 16A). This stable fluctuation suggests that the protein remains in a conformation close to its initial state, with minor deviations. These deviations occur due to inherent flexibility or environmental factors. This indicates that HSA undergoes only minor structural deviations, suggesting that BMBH binding does not significantly alter the overall conformation of HSA. This stability implies that BMBH fits well within its binding pocket without inducing large-scale structural changes.

Conversely, the RMSD of the ligand demonstrates a similar pattern throughout the simulation trajectory. Additionally, the ligand’s RMSD exhibits discernible oscillatory behavior, especially after 20ns of the simulation (Fig. 16B). These steady fluctuation in the ligand’s RMSD suggests a stable binding and interaction within the binding site of HSA, confirming that BMBH remains consistently bound within the binding site without excessive movement or displacement.

The evaluation of the protein backbone’s compactness, assessed through radius of gyration (Rg) analysis, provides insights into the overall structural stability of the complex. The observed low oscillation in Rg, particularly noticeable after about 20 ns of the simulation, suggests high compactness of the protein backbone over time (Fig. 16C). Furthermore, we assessed the solvent-accessible surface area (SASA) of the protein throughout the simulation to gauge its stability in the simulated environment. The SASA measurements corroborate the stability observed in the protein RMSD analysis, showing a consistent oscillation between 315 and 325 nm^2^. This stable oscillation suggests that the protein maintains its exposed surface area to the surrounding solvent molecules without significant perturbations, supporting the notion of a dynamically stable protein–ligand complex complex despite ligand binding (Fig. 16D).

The lack of major fluctuations in RMSD and SASA collectively indicate that BMBH remains securely positioned within its binding pocket throughout the 200 ns simulation. These findings suggest that BMBH-HSA binding is stable over time, supporting its pharmacokinetic relevance in terms of drug transport and bioavailability.

**Fig. 17:**
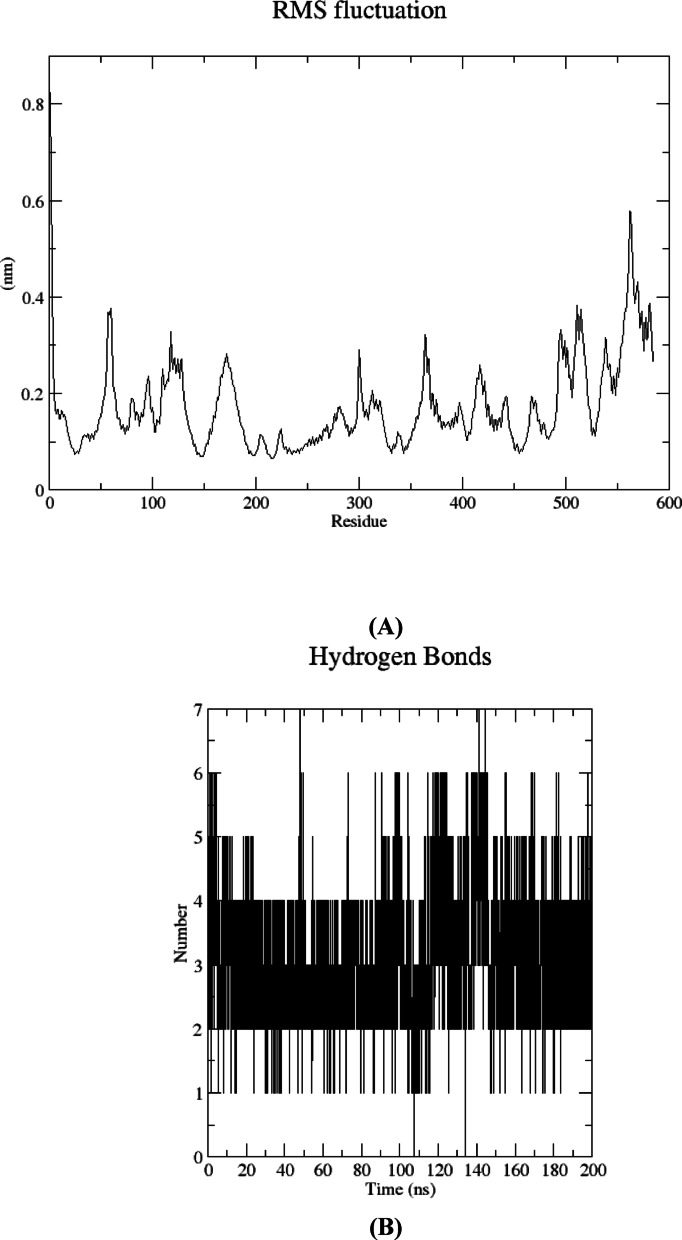
Structural dynamics calculated during the 200 ns of MD trajectories; root mean square fluctuation (RMSF) of protein backbone (**A**), number of H-bonds formed between HSA and BMBH (**B**).

To further evaluate the stability of the complexes, the root mean square fluctuation (RMSF) of the backbone residues was calculated, with the aim of assessing the rigidity and flexibility of residues throughout the 200 ns molecular dynamics (MD) simulation. As illustrated in Fig. 17A, the complex exhibits comparable RMSF patterns, with residues engaged in ligand interactions demonstrating minimal fluctuation (< 0.2 nm), such as Tyr150, Lys199, and Arg222. These residues are aligned with the residues involved in the interaction of BMBH with the HSA binding pocket, as reported in our docking study. Also, the number of H-bonds formed during the 200 ns trajectory confirms that at least 3–4 H-bonds are stable throughout the simulation which aligns with the docking results (Fig. 17B).

## Conclusion

The structural properties of the BMBH compound were confirmed using FT-IR, UV-vis, XRD, and SEM spectra. Computed vibrational frequencies of methyl groups closely matched the experimental FT-IR spectrum. A reduced HOMO-LUMO energy gap suggests charge transfer interactions, indicating NLO activity. Topological analyses (AIM, NBO, ELF, RDG) revealed hydrogen and halogen bonding characteristics, while the MEP map highlighted oxygen and chlorine as regions of negative potential. Mulliken charge analysis showed similar positive charges for all methyl hydrogen atoms. Adsorption studies on a Graphene surface demonstrated effective interactions with the hydrogen of HCl and methyl groups on the electron-rich surface, with a binding energy of − 31.22 kcal/mol, suggesting hydrogen storage potential. A validated docking setup was employed to examine the ligand-receptor interactions and binding mechanisms of bambuterol (binding score = -− 7.99 kcal/mol) with the dementia-related enzyme butyrylcholinesterase, identifying it as a potential inhibitor. This inhibition will lead to increased acetylcholine levels, which is relevant for conditions like Alzheimer’s disease and other neurodegenerative disorders. RMSD, RMSF, radius of gyration, and H-bond parameters exported from molecular dynamics simulation support the notion of a dynamically stable protein-ligand complex.

## Data Availability

The datasets presented in this study can be available from the corresponding author on reasonable request.
